# Evidence for a Direct Harmful Effect of Alcohol on Myocardial Health: A Large Cross‐Sectional Study of Consumption Patterns and Cardiovascular Disease Risk Biomarkers From Northwest Russia, 2015 to 2017

**DOI:** 10.1161/JAHA.119.014491

**Published:** 2019-12-18

**Authors:** Olena Iakunchykova, Maria Averina, Alexander V. Kudryavtsev, Tom Wilsgaard, Andrey Soloviev, Henrik Schirmer, Sarah Cook, David A. Leon

**Affiliations:** ^1^ Department of Community Medicine UIT The Arctic University of Norway Tromsø Norway; ^2^ Department of Laboratory Medicine University Hospital of North Norway Tromsø Norway; ^3^ Department of Innovative Programs Northern State Medical University Arkhangelsk Russia; ^4^ Department of Psychiatry and Clinical Psychology Northern State Medical University Arkhangelsk Russia; ^5^ Department of Cardiology Akershus University Hospital Lørenskog Norway; ^6^ Institute of Clinical Medicine Campus Ahus University of Oslo Norway; ^7^ Department of Clinical Medicine UIT The University of Norway Tromsø Norway; ^8^ Department of Noncommunicable Disease Epidemiology London School of Hygiene and Tropical Medicine London United Kingdom

**Keywords:** alcohol use, CRP (C‐reactive protein), NT‐proBNP (N‐terminal pro‐B‐type natriuretic peptide), troponin T, Cardiovascular Disease, Epidemiology, Risk Factors

## Abstract

**Background:**

Alcohol drinking is an increasingly recognized risk factor for cardiovascular disease. However, there are few studies of the impact of harmful and hazardous drinking on biomarkers of myocardial health. We conducted a study in Russia to investigate the impact of heavy drinking on biomarkers of cardiac damage and inflammation.

**Methods and Results:**

The Know Your Heart study recruited a random sample of 2479 participants from the population of northwest Russia (general population) plus 278 patients (narcology clinic subsample) with alcohol problems. The general population sample was categorized into harmful drinkers, hazardous drinkers, nonproblem drinkers, and nondrinkers, according to self‐reported level of alcohol consumption, whereas the narcology clinic sample was treated as the separate group in the analysis. Measurements were made of the following: (1) high‐sensitivity cardiac troponin T, (2) NT‐proBNP (N‐terminal pro‐B‐type natriuretic peptide), and (3) hsCRP (high‐sensitivity C‐reactive protein). The narcology clinic subsample had the most extreme drinking pattern and the highest levels of all 3 biomarkers relative to nonproblem drinkers in the general population: high‐sensitivity cardiac troponin T was elevated by 10.3% (95% CI, 3.7%–17.4%), NT‐proBNP by 46.7% (95% CI, 26.8%–69.8%), and hsCRP by 69.2% (95% CI, 43%–100%). In the general population sample, NT‐proBNP was 31.5% (95% CI, 3.4%–67.2%) higher among harmful drinkers compared with nonproblem drinkers. Overall, NT‐proBNP and hsCRP increased with increasing intensity of alcohol exposure (test of trend *P*<0.001).

**Conclusions:**

These results support the hypothesis that heavy alcohol drinking has an adverse effect on cardiac structure and function that may not be driven by atherosclerosis.

**Clinical Perspective**

**What Is New?**

- In the population‐based study, we observed elevated levels of markers of heart damage, cardiac wall stretch, and general inflammation among heavy alcohol users compared with nonproblem drinkers.
- Heavy drinking was confirmed as an important risk factor of cardiovascular disease, with probable direct effect on cardiac structure and function.

**What Are the Clinical Implications?**

- Prevention of cardiovascular diseases in the general population should include screening and intervening on harmful and hazardous alcohol use.

## Introduction

Alcohol drinking is increasingly recognized as a risk factor for cardiovascular disease (CVD).^1^ Alcohol, even when consumed in moderation, is associated with complex changes in blood biochemistry, involving changes in many biomarkers for cardiometabolic risk.^2^ Binge drinking is associated with alcoholic cardiomyopathy, high blood pressure, increased risk of myocardial infarction, arrhythmias, and fatal cardiac arrest and stroke.^3^ However, the causal nature of many of the associations between heavy alcohol use and CVD biomarkers as well as the mediation pathways between alcohol use and cardiovascular outcomes are not fully understood. In particular, it is unclear whether any effect is through alcohol's effect on the atherosclerotic process in vessels as distinct from direct toxic damage to the myocardium.

Levels of blood‐based cardiovascular biomarkers can be used as proxy measures of cardiovascular health. High‐sensitivity cardiac troponin T (hs‐cTnT) and NT‐proBNP (N‐terminal pro‐B‐type natriuretic peptide) were both developed for use in clinical cardiology and are now increasingly used in population‐based studies of CVD. When evaluating general population cohorts, any concentration of hs‐cTnT >3 ng/L has been associated with subclinical CVD and has adverse prognostic implications.^4^ Cardiac wall stretch biomarker NT‐proBNP has been mostly used for diagnosis of heart failure and for prognosis in the setting of heart failure.^5^ However, in population‐based samples, low‐grade elevation in NT‐proBNP was shown to be an early marker of cardiac injury that is not yet clinically evident.^6^ Assessment of natriuretic peptides can predict first‐onset heart failure or improve prediction of coronary heart disease in people without known CVD.^7^ In addition, NT‐proBNP concentration predicted stroke as strongly as a diagnosis of coronary heart disease. This could partly be explained by associations between NT‐proBNP concentration and stroke risk factors: left ventricular hypertrophy and atrial fibrillation.^7^, ^8^, ^9^

There has been substantial interest in CRP (C‐reactive protein) as a risk predictor related to the underlying inflammatory nature of atherosclerosis,^10^ although there is evidence that it is in itself not causal but may instead be a marker of a general inflammatory disease process.^11^ A 1000‐fold elevation of CRP is indicative of acute inflammation,^12^ whereas lower persistent elevation of hsCRP (high‐sensitivity C‐reactive protein) may be caused by low‐grade systemic inflammatory processes associated with atherosclerosis.^13^ Increased levels of hsCRP have been predictive of future cardiovascular events^14^, ^15^ and have been associated with coronary plaque burden.^16^

Previous research has looked at the relationship of biomarkers with classic risk factors for CVD, among them smoking, obesity indexes, blood pressure, and lipid profiles.^13^, ^17^, ^18^, ^19^, ^20^ However, there has been little work on the association of alcohol with biomarkers of heart damage, cardiac wall stretch, and systemic low‐grade inflammation. Most of the published work was done in the populations with relatively moderate levels of alcohol consumption,^18^, ^21^, ^22^, ^23^ with the exception of one study that showed prospectively an association between heavy drinking and heart failure in vulnerable men with underlying myocardial ischemia.^24^ As noted elsewhere, there is a gap in the research literature on heavy drinking patterns affecting cardiovascular outcomes.^25^

Russia is one of the countries that has had a tradition of heavy drinking of spirits and has been characterized as having a particularly harmful drinking profile.^1^ Studies of CVD biomarkers in the Russian population make it possible to achieve 2 goals: (1) to investigate the mechanisms by which hazardous and harmful patterns of alcohol use increase the risk of cardiovascular outcomes and (2) to help clarify the role of heavy alcohol use in explaining why Russia has one of the highest CVD rates of any country.^26^ In this study, we used measures of hs‐cTnT, NT‐proBNP, and hsCRP to assess the damage to myocardium, cardiac wall stretch, and general low‐grade inflammation in heavy drinking individuals recruited through state‐run facilities for treatment of alcohol use disorders and in a large general population sample in Russia categorized according to level and pattern of alcohol use.

## Methods

Requests to access the data set from bona fide researchers may be sent to the International Project on Cardiovascular Disease in Russia.^27^

### Study Design

The Know Your Heart study recruited 2479 participants from the general population of the city of Arkhangelsk in northwest Russia from 2015 to 2018. A detailed account of the rationale and description of the methods of the study has been published previously.^28^ At the same time, we recruited 278 patients from the Arkhangelsk Regional Psychiatric Hospital with a primary diagnosis of alcohol problems.^28^ The latter group is referred to subsequently as the narcology clinic subsample, consistent with Russian terminology. The study sample was almost exclusively of European descent.

### Ethical Approval

All procedures performed were in accordance with the ethical standards of the institutional research committee (ethics committees of the London School of Hygiene and Tropical Medicine [London, UK] and the Northern State Medical University [Arkhangelsk, Russia]) and with the 1964 Declaration of Helsinki and its later amendments or comparable ethical standards. All participants included in the analysis gave signed informed consent.

### Study Participants

The general population sample from Arkhangelsk was recruited at random (stratified by age, sex, and district of residence) using the regional health insurance fund register as the sampling frame. Trained interviewers visited the addresses selected and invited the appropriate resident at each address to take part in the study. A minimum of 3 attempts were made to get a response from each address. When successful, an interview was conducted about circumstances, health, and behaviors of the participant. The response rate was 68% of the addresses where contact with a person of the target age and sex was established, and 96% of those interviewed took part in a subsequent health check.^28^

In addition, a sample of heavy drinkers (narcology clinic subsample) were recruited from inpatients at the regional psychiatric hospital. The inclusion criteria were as follows: age of 35 to 69 years, resident in the city of Arkhangelsk or Arkhangelsk region, and admitted to the narcological department of the regional psychiatric hospital with a primary diagnosis related to alcohol drinking. People with ≥1 of the following characteristics were excluded:

1. Experiencing alcohol withdrawal symptoms or during the first week of alcohol detoxification;
2. Behavior that suggested that an individual could pose a threat to the safety of the clinic staff or other participants during the survey;
3. Current or past misuse of drugs other than nicotine or alcohol;
4. Unable to give informed consent for participation in the study (eg, severe cognitive deficit or acute psychiatric illness).

Clinicians at the hospital used their judgement to decide which participants should or should not be invited. Signed informed consent was obtained. A total of 278 patients were recruited of 322 patients invited (85.4%).

We analyzed data on 2354 participants from the general population in Arkhangelsk plus 271 individuals from the narcology clinic subsample who attended the health check and for whom blood analyte concentrations were available.

### Data and Sample Collection

The baseline interview was administered by a trained interviewer using a tablet computer‐assisted personal interviewing device. For the general population sample, the interview was done in people's homes in nearly all cases. For the narcology clinic subsample, it was done at the Arkhangelsk Regional Psychiatric Hospital by the same set of trained interviewers. Information was collected on medical history and socioeconomic circumstances, education, and lifestyle.

The subsequent health check comprised a physical examination (including blood pressure, height, waist and hip circumference, and weight) and blood sample collection. Participants were requested not to eat or drink alcohol in the 4 hours before their appointment. Participants in the narcology clinic subsample were transported to the research clinic for the health check, accompanied by a nurse. A second interview was conducted at this stage that recorded medical history, use of medications, alcohol use, and smoking.

A total of 50 mL of blood was taken from each participant. Samples were centrifuged, and serum was transferred to barcoded 1.8‐mL cryovials and frozen (−80°C) within 2 hours after venipuncture. These were subsequently shipped to a laboratory in Moscow, where they were stored at −80°C and then analyzed in a single batch at the end of the fieldwork. The laboratory staff were blind to all characteristics of participants, including whether serum was from the narcology clinic subsample or the general population sample.

### Outcome Variables

hs‐cTnT and NT‐proBNP were measured using a high‐sensitivity electrochemiluminescence immunoassay (Roche Diagnostics GmbH, Hitachi, Japan) on a Cobas e411 analyzer. hsCRP was measured using a high‐sensitivity immunoturbidimetric test on AU 680 Chemistry System Beckman Coulter. The lower limit of detection for hs‐cTnT test was 3 ng/L, and 54 participants (2.07%) with values below the limit of detection had their values recoded to 2.9 ng/L. The limit of detection of NT‐proBNP test was 5 ng/L, and NT‐proBNP values of 19 participants (0.7%) with values below the limit of detection were recoded to 4.9 ng/L. Because we were interested in low‐grade inflammation that is not caused by acute infection, 38 participants with hsCRP values >99th percentile for the general population (30 mg/L) were excluded from the statistical analysis of hsCRP.

### Exposure Variables

We defined 2 categorical exposure variables. The first was binary and divided the study group into those from the narcology clinic subsample and those from the general population sample. The second exposure variable further categorized the general population sample into groups based on self‐report of various dimensions of alcohol consumption. Those who reported not drinking alcoholic beverages during the past 12 months at baseline interview and health check were classified as nondrinkers. The categories of harmful and hazardous drinkers were defined using 3 instruments: the validated Russian‐language translations of the Alcohol Use Disorders Identification Test,^29^ the Cut down, Annoyed, Guilty, Eye Opener (CAGE) instrument,^30^ and questions on alcohol drinking pattern previously found to be highly predictive of mortality in Russia.^31^, ^32^ The Alcohol Use Disorders Identification Test is a screening test for hazardous and harmful drinking, whereas CAGE is used to screen for alcoholism in clinical settings. The CAGE score was adapted to have a reference period of the past 12 months rather than ever in a participant's lifetime, in keeping with a previous study from Russia because of interest in alcohol use in the recent past.^33^ The scheme for categorizing the general population sample into 4 groups is presented in the Figure.

**Figure 1:**
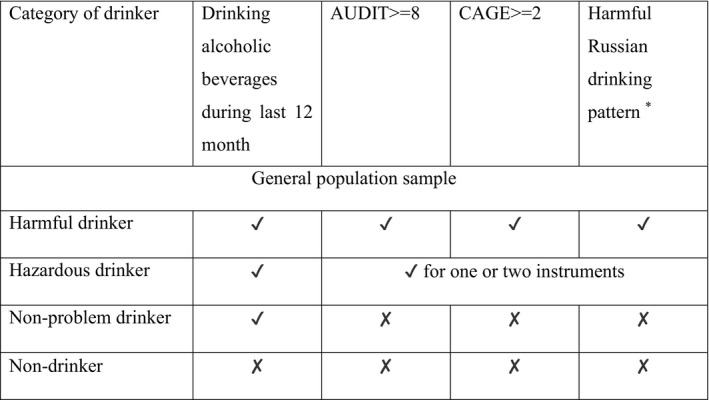
The assignment scheme of the general population sample into categories by drinking status: (1) harmful drinkers, (2) hazardous drinkers, (3) nonproblem drinkers, and (4) nondrinkers. *Twice weekly or more frequency of hangover and/or excessive drunkenness and/or sleeping in clothes at night because of drunkenness and/or failing their family or personal obligations because of drinking and/or drinking nonbeverage alcohols (sources of ethanol not intended for drinking, such as medicinal tinctures) and/or ≥1 episodes of zapoi (a period of ≥2 days of being drunk, during which a participant is withdrawn from normal social life).^32^ AUDIT indicates Alcohol Use Disorders Identification Test; GAGE, Cut down, Annoyed, Guilty, Eye Opener.

To validate the approach chosen for classification of the general population sample into drinking categories, we used the following: (1) information on alcohol volume consumed during the past 12 months, calculated using the standard quantity frequency approach^34^; (2) history of asking for help with alcohol problems from social workers or physicians; and (3) blood biomarkers of alcohol use. γ‐Glutamyl transferase and carbohydrate‐deficient transferrin (CDT) are biomarkers of excessive drinking.^35^ We used a previously developed approach^36^ to calculate a combined biomarker value of ɣ‐%CDT=[0.8×ln(γ‐glutamyl transferase)]+[1.3×ln(%CDT)], with a cutoff value of 4.0 for heavy drinking. γ‐Glutamyl transferase was measured in all study participants. Because of cost, CDT was not assayed in everyone. It was measured in all 271 patients receiving treatment for alcohol problems, all 400 problem drinkers (Alcohol Use Disorders Identification Test score ≥8 or CAGE score ≥2), all 143 nonproblem drinkers drinking >5 L per year, and 244 randomly selected nondrinkers and nonhazardous drinkers. The combined ɣ‐%CDT was thus only available for 1032 participants. As this biomarker was only used to establish the face validity of the alcohol categorization, the fact that it was only available for a subset of study subjects did not affect the numbers used in the main analyses.

### Other Covariates

Information was available on classic risk factors, including those that are on the potential causal pathway between alcohol use and CVD biomarkers. We constructed directed acyclic graphs to identify the minimal sufficient adjustment set of variables for estimating the total effect of alcohol use (Figures S1 through S3). These were age, sex, smoking, and education. Education was classified into 4 categories: incomplete secondary or lower; secondary or professional school; incomplete higher or specialized secondary (eg, medical, teacher training college, or technical); and higher (university). Professional schools include institutions that provide professional training but no degree. Smoking status was categorized as current smokers, ex‐smokers, and never smokers. For current smokers, the number of cigarettes smoked was specified as 1 to 10/day, 11 to 20/day, and >20/day.

Other variables used in the analysis included potential mediators. These included systolic and diastolic blood pressure (mean of second and third measurements) and use of antihypertensives, determined according to recorded medications coded to the Anatomical Therapeutic Chemical classification as C02 (antihypertensives), C03 (diuretics), C07 (β‐blocking agents), C08 (calcium channel blockers), or C09 (agents operating on the renin‐angiotensin system). A small proportion of participants self‐reported use of blood pressure–lowering medication without a corresponding Anatomical Therapeutic Chemical code being found. These participants were also defined as being on antihypertensives.

Body mass index (BMI) was calculated as weight (in kilograms) divided by height (in meters) squared. Waist/hip ratio was the mean of 2 measurements of waist divided by the mean of 2 measurements of hip. The blood lipid profile was measured and included total cholesterol, low‐density lipoprotein cholesterol, high‐density lipoprotein cholesterol, apolipoprotein A1, and apolipoprotein B. Renal function was assessed by measuring cystatin C and estimated glomerular filtration rate using the Chronic Kidney Disease Epidemiology Collaboration cystatin C equation.^37^

### Statistical Analyses

Descriptive tabulations of participant characteristics were age and sex standardized to the Standard European Population 2013. hs‐cTnT, NT‐proBNP, and hsCRP had right‐skewed distributions and were ln transformed for analysis.

We assessed the association between alcohol use and biomarkers of CVD (hs‐cTnT, NT‐proBNP, and hsCRP) by comparing geometric mean levels of biomarkers in the narcology and general population samples. Next, we compared means of ln‐transformed biomarkers across the categories of alcohol consumption: (1) narcology clinic subsample; (2) general population sample, harmful drinking pattern; (3) general population sample, hazardous drinking pattern; (4) general population sample, nonproblem drinkers; and (5) general population sample, nondrinkers. This approach allowed a nuanced assessment of CVD biomarkers, depending on the drinking pattern, separating nonproblem drinkers as a comparison group and determining if there was an increasing trend with increased intensity of alcohol use.

The sociodemographic characteristics and CVD risk factors were compared by the categories of main exposure variable using heterogeneity tests adjusting for age and sex in generalized linear models. The associations between alcohol use and ln‐transformed biomarkers of CVD (hs‐cTnT, NT‐proBNP, and hsCRP) were assessed using multivariable adjusted linear regression models. Age was included in the model as a continuous variable. Quadratic and cubic terms were added to account for nonlinearity and kept in the modelif associated with an outcome at *P*<0.05. Model 1 involved adjustment for age and sex. Model 2 adjusted for potential confounders (age, sex, smoking, and education). Model 3 additionally included possible mediators (waist/hip ratio, BMI, lipids [low‐density lipoprotein, high‐density lipoprotein, and apolipoprotein B/apolipoprotein A1 ratio], blood pressure [systolic and diastolic], use of blood pressure medication, and estimated glomerular filtration rate). This final model provided an estimate for the direct effect of alcohol on cardiac damage, cardiac wall stretch, and low‐grade inflammation. A test for increasing linear trend in means of biomarkers across the categories of alcohol exposure was done with *df*=1. To make the regression coefficients more interpretable and comparable, they were back transformed and presented as percentage of difference in mean compared with the reference category (nonproblem drinkers). Statistical analysis was performed using SAS software 9.4 (SAS Institute Inc, Cary, NC).

## Results

The descriptive characteristics of the narcology clinic subsample and the general population sample (age and sex standardized) are presented in Table 1. The average age of the narcology clinic subsample was 48.5 years and that of the general population sample was 53.7 years. The narcology clinic sample was 76.8% men, and the general population sample was 41.7% men. On average, the narcology clinic subsample had lower systolic blood pressure (potentially because of clinical management during hospital admission), lower low‐density lipoprotein and total cholesterol values, lower BMI and waist circumference, and lower estimated glomerular filtration rate compared with the general population sample. A much higher proportion of narcology clinic subsample compared with the general population sample were current smokers. Detectable hs‐cTnT was observed in 98% of participants, whereas the equivalent figure for NT‐proBNP was 99%. The geometric means for hs‐cTnT, NT‐proBNP, and hsCRP were significantly higher in the narcology clinic subsample compared with the general population sample.

**Table 1:** Age‐ and Sex‐Standardized Means and Proportions With 95% Confidence Intervals (n=2625)

Variables	Narcology Clinic Subsample (n=271)	General Population Sample (n=2354)	*P* Value, Test of Heterogeneity
Current drinkers^a^	1.00	0.91 (0.89–0.92)	<0.001
Harmful Russian drinking pattern^b^	0.89 (0.85–0.93)	0.08 (0.07–0.09)	<0.001
AUDIT score ≥8	0.95 (0.90–1.00)	0.16 (0.15–0.18)	<0.001
CAGE score ≥2	0.92 (0.87–0.98)	0.15 (0.14–0.16)	<0.001
Current smoking	0.75 (0.68–0.82)	0.26 (0.25–0.28)	<0.001
Use of antihypertensive medication	0.33 (0.26–0.40)	0.39 (0.37–0.41)	0.102
BMI, mean, kg/m^2^	25.3 (24.5–26.2)	27.6 (27.3–27.8)	<0.001
Waist/hip ratio, mean	0.90 (0.89–0.91)	0.89 (0.88–0.89)	0.028
Waist, mean, cm	86.9 (84.8–88.9)	91.2 (90.7–91.7)	<0.001
Systolic blood pressure, mean, mm Hg	127 (124–130)	132 (131–132)	0.006
Diastolic blood pressure, mean, mm Hg	83.6 (81.8–85.4)	83.5 (83.1–84.0)	0.95
Total cholesterol, mean, mmol/L	5.15 (4.98–5.32)	5.37 (5.33–5.42)	0.012
LDL cholesterol, mean, mmol/L	3.43 (3.29–3.57)	3.63 (3.60–3.67)	0.006
HDL cholesterol, mean, mmol/L	1.44 (1.38–1.50)	1.43 (1.42–1.45)	0.891
Apolipoprotein B/apolipoprotein A1 ratio, mean	0.71 (0.67–0.74)	0.72 (0.72–0.73)	0.353
eGFR (cystatin C), mean, mL/min per 1.73 m^2^	74.1 (72.0–76.1)	80.1 (79.6–80.6)	<0.001
hs‐cTnT, GM, ng/L	7.09 (6.63–7.58)	6.43 (6.32–6.54)	0.006
NT‐proBNP, GM, pg/mL	112 (95.7–131)	72.6 (69.7–75.6)	<0.001
hsCRP, GM, mg/L	3.06 (2.55–3.68)	1.51 (1.44–1.58)	<0.001
Triglycerides, GM, mmol/L	1.37 (1.25–1.50)	1.24 (1.21–1.27)	0.043

Data are standardized to the standard European population 2013. AUDIT indicates Alcohol Use Disorders Identification Test; BMI, body mass index; eGFR, estimated glomerular filtration rate; GM, geometric mean; HDL, high‐density lipoprotein; hsCRP, high‐sensitivity C‐reactive protein; hs‐cTnT, high‐sensitivity cardiac troponin T; LDL, low‐density lipoprotein; NT‐proBNP, N‐terminal pro‐B‐type natriuretic peptide; CAGE, Cut down, Annoyed, Guilty, Eye Opener.

a All participants from the narcology clinic sample are current drinkers, but they were not drinking during the period of admission to the narcology clinic.

b Twice weekly or more frequency of hangover and/or excessive drunkenness and/or sleeping in clothes at night because of drunkenness and/or failing their family or personal obligations because of drinking and/or drinking nonbeverage alcohols (sources of ethanol not intended for drinking, such as medicinal tinctures) and/or ≥1 episodes of zapoi (a period of ≥2 days of being drunk, during which a participant is withdrawn from normal social life).

The face validity of our categorization of alcohol use is demonstrated in Table 2. This shows indicators of drinking for each of the drinking categories derived from self‐reported alcohol use in the general population and the narcology clinic sample. Almost all of the alcohol measures show a clear trend across the categories. The concentration of alcohol use biomarkers (CDT and γ‐glutamyl transferase) and the proportion of the participants with an elevated combined biomarker of alcohol use are highest in harmful drinkers, intermediate in hazardous drinkers, and lowest in nonproblem drinkers. Similarly, the volume of alcohol consumed by drinkers during the past year and the amount of alcohol consumed per day are highest in harmful drinkers, intermediate in hazardous drinkers, and lowest in nonproblem drinkers. Of the 227 nondrinkers, 82 were former drinkers, as distinct from life‐long nondrinkers.

**Table 2:** Descriptive Measures of Alcohol Use by Categories of Alcohol Use

Variables	Narcology Clinic Sample (n=271)	General Population Sample				*P* Value^b^
		Harmful Drinkers (n=71)	Hazardous Drinkers (n=424)	Nonproblem Drinking (n=1632)	Nondrinkers^a^ (n=227)	
Combined biomarker of heavy alcohol use (GGT and CDT) ≥4, N (%)	135 (50.9)	27 (38.6)	55 (14.0)	23 (5.2)	0	<0.001
Have asked for help of narcologist or social worker for drinking problem, N (%)	271 (100)	26 (36.6)	27 (6.4)	12 (0.8)	19 (23.2)^d^	<0.001
Drinking >40 g of alcohol per day, N (%)^c^	62 (23.7)	26 (36.6)	48 (11.3)	12 (0.7)	0	<0.001
Binge drinking (60 g of alcohol per drinking occasion) at least once a month, N (%)	189 (70.5)	49 (69.0)	215 (51.9)	76 (4.8)	0	<0.001
Alcohol consumed per year, mean, L^c^	15.0	19.0	8.5	1.9	0	<0.001
Alcohol consumed per day, mean, g^c^	33.45	40.09	18.41	4.04	0.00	<0.001
GGT, U/L	68.02	44.39	38.48	25.03	23.69	<0.001
CDT, %	1.64	1.60	0.94	0.74	0.53	<0.001

CDT indicates carbohydrate‐deficient transferrin; GGT, γ‐glutamyl transferase.

a Nondrinkers include lifetime abstainers and ex‐drinkers.

b Test for linear trend, adjusted for age and sex.

c Alcohol consumption recorded for the past 12 months.

d Among ex‐drinkers.

### CVD Biomarkers in the Narcology Clinic Subsample Versus the General Population

After adjustment for age, sex, smoking, and education, the levels of all biomarkers (hs‐cTnT, NT‐proBNP, and hsCRP) were higher in the narcology clinic subsample compared with the general population sample as a whole (Table 3). Specifically, hs‐cTnT was higher by 12.3% (95% CI, 5.9%–19.1%) and NT‐proBNP was higher by 43.9% (95% CI, 25.4%–65.1%), whereas hsCRP was higher by 66.0% (95% CI, 41.7%–94.5%).

**Table 3:** Percentage Differences in hs‐cTnT, NT‐proBNP, and hsCRP Between Narcology Clinic Subsample and General Population

Narcology Clinic Subsample vs General Population	% Difference (95% CI), Adjusted for Age and Sex	% Difference (95% CI), Additionally Adjusted for Smoking and Education	% Difference (95% CI), Additionally Adjusted for Mediators^a^
hs‐cTnT (n=2595)	8.2 (2.6–14.3)	12.3 (5.9–19.1)	12 (5.7–18.7)
NT‐proBNP (n=2595)	63.3 (43.8–85.5)	43.9 (25.4–65.1)	30.9 (14.6–49.6)
hsCRP (n=2562)	107.2 (78.8–140.2)	66.0 (41.7–94.5)	98.3 (71.2–129.8)

Dependent variable was ln transformed, and the regression coefficients were back transformed and presented as percentage difference in mean in comparison to the reference group. hsCRP indicates high‐sensitivity C‐reactive protein; hs‐cTnT, high‐sensitivity cardiac troponin T; NT‐proBNP, N‐terminal pro‐B‐type natriuretic peptide.

a Possible mediators included were systolic and diastolic blood pressure, use of blood pressure medication, lipid profile (total cholesterol, low‐density lipoprotein cholesterol, high‐density lipoprotein cholesterol, apolipoprotein A1, and apolipoprotein B), renal function (estimated glomerular filtration rate), body mass index, and waist/hip ratio.

### CVD Biomarkers Across 5 Categories of Alcohol Use

Consistent with the previous analysis, compared with nonproblem drinkers in the general population sample, the narcology clinic subsample had much higher levels of hs‐cTnT, NT‐proBNP, and hsCRP (Table 4).

**Table 4:** Percentage Differences in hs‐cTnT, NT‐proBNP, and hsCRP Between Levels of Alcohol Use

Alcohol Use	% Difference (95% CI), Adjusted for Age and Sex	% Difference (95% CI), Additionally Adjusted for Smoking and Education	% Difference (95% CI), Additionally Adjusted for Mediators^a^
hs‐cTnT (n=2595)			
Narcology clinic subsample	6.6 (0.7 to 12.8)	10.3 (3.7 to 17.4)	10.3 (3.7 to 17.3)
Harmful drinkers, general population sample	−14.4 (−22.6 to −5.3)	−11.5 (−20.1 to −2)	−9.6 (−18.2 to −0.1)
Hazardous drinkers, general population sample	−3.3 (−7.7 to 1.4)	−2.6 (−7.1 to 2.1)	−1.8 (−6.3 to 2.8)
Nonproblem drinking, general population sample	0 (Reference group)	0 (Reference group)	0 (Reference group)
Nondrinkers, general population sample	2.1 (−3.6 to 8.2)	1.6 (−4.1 to 7.7)	−0.6 (−6.1 to 5.1)
*P* value for linear trend (among drinkers)	0.272	0.068	0.047
*P* value for heterogeneity	<0.001	<0.001	<0.001
NT‐proBNP (n=2595)			
Narcology clinic subsample	68.6 (47.6 to 92.6)	46.7 (26.8 to 69.8)	34.9 (17.1 to 55.4)
Harmful drinkers, general population sample	45.6 (14.9 to 84.6)	31.5 (3.4 to 67.2)	30.1 (3.5 to 63.5)
Hazardous drinkers, general population sample	1.5 (−9.1 to 13.3)	−3.5 (−13.7 to 7.8)	1.9 (−8.4 to 13.3)
Nonproblem drinking, general population sample	0 (Reference group)	0 (Reference group)	0 (Reference group)
Nondrinkers, general population sample	10.3 (−3.7 to 26.4)	6.6 (−6.9 to 22.1)	0.9 (−11.3 to 14.8)
*P* value for linear trend (among drinkers)	<0.001	<0.001	<0.001
*P* value for heterogeneity	<0.001	<0.001	<0.001
hsCRP (n=2562)			
Narcology clinic subsample	117.1 (86.1 to 153.2)	69.2 (43 to 100.2)	99.7 (70.9 to 133.4)
Harmful drinkers, general population sample	33.9 (2.2 to 75.4)	13.4 (−13.7 to 49)	28.4 (0.2 to 64.6)
Hazardous drinkers, general population sample	14.7 (1.1 to 30.2)	6.9 (−5.8 to 21.4)	0.5 (−10.5 to 12.9)
Nonproblem drinking, general population sample	0 (Reference group)	0 (Reference group)	0 (Reference group)
Nondrinkers, general population sample	−9 (−22.1 to 6.4)	−12.2 (−24.8 to 2.5)	−10.7 (−22.4 to 2.8)
*P* value for linear trend (among drinkers)	<0.001	<0.001	<0.001
*P* value for heterogeneity	<0.001	<0.001	<0.001

Dependent variable was ln transformed, and the regression coefficients were back transformed and presented as percentage difference in mean in comparison to the reference group. hsCRP indicates high‐sensitivity C‐reactive protein; hs‐cTnT, high‐sensitivity cardiac troponin T; NT‐proBNP, N‐terminal pro‐B‐type natriuretic peptide.

a Possible mediators included were systolic and diastolic blood pressure, use of blood pressure medication, lipid profile (total cholesterol, low‐density lipoprotein cholesterol, high‐density lipoprotein cholesterol, apolipoprotein A1, and apolipoprotein B), renal function (estimated glomerular filtration rate), body mass index, and waist/hip ratio.

hs‐cTnT was elevated by 10.3% (95% CI, 3.7%–17.4%) in the narcology clinic subsample compared with the nonproblem drinkers in the general population, controlling for sex, age, smoking, and education. However, hs‐cTnT levels were lower in the group of harmful drinkers in the general population compared with nonproblem drinkers. Adjustment for the additional set of variables that are likely to be the mediators of the association between extremely heavy alcohol use and cardiac injury (determined via hs‐cTnT) had only a minor effect on parameter estimates (Table 4).

Harmful drinkers in the general population had an elevated concentration of NT‐proBNP by 31.5% (95% CI, 3.4%–67.2%) compared with nonproblem drinkers, but to lesser extent than in the narcology clinic subsample (46.7%; 95% CI, 26.8%–69.8%), controlling for age, sex, smoking, and education. Adjustment for potential mediators of the association between excessive alcohol use and cardiac wall stretch (measured by NT‐proBNP) resulted in some attenuation of the effect estimate (Table 4).

The elevation of low‐grade systemic inflammation marker hsCRP by 69.2% (95% CI, 43%–100%) was observed in the narcology clinic subsample compared with nonproblem drinkers in the general population sample, controlled for age, sex, smoking, and education. Intermediate elevations were also seen for harmful drinkers. Further adjustment for covariates that are likely to be on the mediation pathway between alcohol use and hsCRP leads to increases in the regression coefficient (Table 4).

Although we did not observe increased levels of cardiac biomarkers in the group of hazardous drinkers in the general population, the trend test across all drinking categories (excluding nondrinkers) was significant for NT‐proBNP and hsCRP, with concentration of biomarkers higher with higher level of alcohol exposure (Table 4).

### Sensitivity Analysis

hs‐cTnT assays are known to show appreciable imprecision at the low values seen in the general population.^38^ The interassay coefficient of variation for values below the limit of quantification (13 ng/L) was 15%. To ensure the robustness of conclusions about hs‐cTnT, we conducted a sensitivity analysis using logistic regression, with hs‐cTnT categorized into values below and above the top quintile in the general population sample (9.34 ng/L). The results of this analysis were consistent with analyses presented above (Table S1).

Separating the category of nondrinkers into never drinkers and ex‐drinkers for regression analysis did not reveal any specific differences in biomarkers between these 2 groups; therefore, the results were presented keeping current nondrinkers as one group.

In a further sensitivity analysis, we excluded those with previous myocardial infarction, operations on the heart, and grade 2 angina (N=307 [11.73%]) to see if elevated cardiac injury and cardiac wall stretch biomarkers were secondary to coronary heart disease. This had no material effect on the associations observed.

## Discussion

In this study, we have shown that markers of cardiac injury hs‐cTnT, cardiac wall stretch NT‐proBNP, and general inflammation hsCRP are substantially elevated among those receiving treatment for alcohol problems at the narcology clinic compared with the general population. Most important, there was a significant linear increasing trend of NT‐proBNP across 4 groups of drinkers: nonproblem drinkers, hazardous drinkers, harmful drinkers, and the narcology clinic sample. Similarly, there was a linear increase in hsCRP levels over drinking groups.

NT‐proBNP has been developed and primarily used in the clinical contexts of diagnosis and prognosis of heart failure.^5^ However, in population‐based samples, low‐grade elevation in NT‐proBNP was shown to be an early marker of cardiac injury that is not yet clinically evident.^6^, ^39^ In this study, we showed markedly elevated levels of NT‐proBNP in the sample receiving treatment at a narcology clinic and intermediate elevation in the general population sample of harmful drinkers. This is consistent with a previous report from the Izhevsk family study and the Belfast (UK) component of the PRIME (Prospective Epidemiological Study of Myocardial Infarction) study that showed elevated NT‐proBNP in hazardous drinkers.^40^ This finding is further supported by increased risk of heart failure among heavy drinking men in the prospective BRHS (British Regional Heart Study).^24^ However, our study goes further by showing that there is a biomarker dose‐response effect across the 4 categories of heavy drinking at levels of NT‐proBNP that are below clinical thresholds for heart failure. Other studies of this question that had inconsistent findings were limited by the fact that the populations they studied had much lower levels of alcohol consumption.^18^, ^21^, ^23^

After the adjustment for the possible mediators of the association between alcohol use and NT‐proBNP (blood pressure, blood lipid indexes, BMI, and kidney function), the regression coefficients were partly attenuated. This could be explained by an effect of alcohol on kidney function because it was shown that risk of chronic kidney disease is higher in people with alcohol use disorder.^41^ Increased blood pressure caused by heavy alcohol use^42^, ^43^ may be responsible for a decrease in kidney function and may lead to hypertensive cardiac injury. Low BMI and altered lipid metabolism in the narcology clinic subsample, caused by alcoholic malnutrition, poor diet, and effects of alcohol, may further contribute to the damage of the myocardium.

The direct toxic effect of alcohol on the heart as a result of persistent heavy drinking is an established mechanism of alcoholic cardiomyopathy.^44^ The condition can be undiagnosed, interact with the atherosclerotic damage to cardiovascular system, and increase the risk of sudden cardiac death.^1^ Estimates of the risk of diagnosed alcoholic cardiomyopathy in people with alcohol use disorders varied between 1% and 40%, depending on the patient population studied.^44^ The observed increasing trend in NT‐proBNP levels across 4 categories of alcohol exposure in our study gives support to the hypothesis that heavy drinking causes subclinical nonischemic myocardial damage. The association of heavy alcohol use with NT‐proBNP in our study is consistent with previous reports of increased NT‐proBNP in left ventricular hypertrophy, atrial fibrillation, and stroke.^7^, ^8^, ^9^

Cardiac troponin T elevation is a biomarker used for the diagnosis of acute myocardial infarction. Development of the testing technology and introduction of hs‐cTnT tests led to recognition that low‐grade elevations of hs‐cTnT are predictive of future cardiovascular events and death in general population.^4^, ^6^, ^45^ Detectable hs‐cTnT is observed in a sizable proportion of individuals without diagnosis of CVD.^18^ In our study, detectable levels of hs‐cTnT were observed in 98% of the study sample, which is higher than in some other population‐based cohorts,^6^, ^46^ but comparable to others.^19^ It has been suggested that long‐term elevation of hs‐cTnT is explained to a greater extent by indexes of heart failure (eg, higher left ventricular mass and lower left ventricular ejection fraction) and increased NT‐proBNP levels than indexes of atherosclerosis or ischemia.^46^, ^47^ Also, hs‐cTnT has been found to be a direct marker of ongoing myocardial fibrosis.^48^ Similarly, in our sample of heavy drinkers at the narcology clinic, elevation of hs‐cTnT may indicate nonischemic injury to the myocardium that occurred because of exposure to high doses of alcohol or its metabolites, such as acetaldehyde. After adjustment for possible mediators (blood pressure, blood lipid indexes, BMI, and kidney function), the regression coefficients for the relationship between heavy alcohol use and hs‐cTnT did not change. Therefore, the effect of heavy alcohol consumption on the myocardium may be explained by the direct injury of myocardium by alcohol or its metabolites that leads to cell death. However, it is unclear why the group of harmful drinkers in the general population have lower levels than nonproblem drinkers, although the CIs around the estimates of effect for hs‐cTnT are large. A literature search for other studies that looked at association between alcohol consumption and hs‐cTnT identified only reports with relatively moderate level of alcohol use, which reported either decreased or the same hs‐cTnT levels in some groups of drinkers compared with nondrinkers.^18^, ^21^, ^22^, ^23^ The reasons for this phenomenon may lie in low precision of hs‐cTnT at low levels, unknown factors influencing the performance of the test, and selection of the comparison group for analysis by categories of alcohol consumption.

A U‐shaped relation between alcohol intake and CRP was previously observed, with heavy drinkers showing higher CRP than moderate drinkers.^49^, ^50^ The elevation of CRP in our study in the narcology clinic sample and the trend for elevated hsCRP across harmful and hazardous drinkers in the general population may have several explanations. The low‐grade elevation of hsCRP in individuals with no overt disease is nonspecific and may reflect exposure to proinflammatory influences, including smoking, particulate air pollutants, aspects of diet, medications, obesity, and the metabolic syndrome.^13^ Although we made efforts to adjust for many of these factors, there is still a strong possibility of residual confounding. Beyond this, there are several explanations for increased hsCRP in the narcology clinic subsample, including, but not limited to, toxic effects of alcohol and its metabolites on the liver, ranging from fatty liver to steatosis, process of detoxification, and exposure to the specific medications during treatment for alcohol problems. Finally, elevated levels of hsCRP in the narcology clinic subsample and the trend across categories of drinkers in the general population may be secondary to an atherosclerotic process facilitated by harmful and hazardous drinking. Thus, it is not possible from our study to determine which of these various explanations may account for the association of hsCRP with harmful and hazardous patterns of drinking.

### Strengths and Limitations

Our study has several strengths that make it a significant contribution to the body of evidence on detrimental effects of alcohol on cardiovascular health by relating harmful and hazardous alcohol use to the markers of cardiac damage, cardiac wall stretch, and low‐grade inflammation. We were able to recruit a substantial number of participants with substantial variation in the level and intensity of alcohol drinking. In addition, this study has addressed a gap in the research literature about the impact of high levels of exposure on cardiac injury^44^ in a country where this exposure is relatively common^25^ and rates of CVD mortality are among the highest in the world.^26^

Previous studies of association between alcohol consumption and CVD biomarkers are few and done in populations with relatively moderate quantities of consumed alcohol, whereas this study was able to differentiate between different patterns of heavy alcohol use, including extreme drinking patterns commonly observed in patients treated in Russian state‐run narcology services. Although patients there may experience multiple comorbidities, they are most likely the consequence of an extremely heavy pattern of alcohol use. Their admission to the narcology clinic was because of the need for detoxification rather than organic comorbidities. Therefore, it is a good setting to study the mechanisms of cardiac injury caused by heavy alcohol use; and the results are likely to be generalizable to all countries and populations.

A particular strength of our study is that the collection of detailed questionnaire information on patterns and quantities of alcohol consumption during the past year has allowed us to construct a plausible grouping of general population participants according to degree of harmfulness of their alcohol consumption with strong face validity. Moreover, our ability to separate nondrinkers from nonproblem drinkers and the availability of data on confounders beyond age and sex have minimized the bias common to many other studies on alcohol drinking and CVD.^51^ We cannot fully exclude the possibility of some residual confounding by smoking that was measured by self‐report. Acknowledging the limitation of cross‐sectional studies to show the direction of the association, we look forward to the prospective studies that may give better support for our conclusions.

## Conclusions

The elevated levels of both NT‐proBNP and hs‐cTnT in the group of extremely heavy drinkers from the narcology subsample are consistent with heavy alcohol drinking leading to nonischemic damage of the heart. Elevated NT‐proBNP in harmful drinkers from the general population provides further evidence for this. Furthermore, exclusion of individuals who had a previous diagnosis of coronary heart disease did not have an impact on the substantive results. However, this study cannot definitively exclude that heavy drinking may also contribute to increased CVD risk through ischemic pathways, as could be indicated by elevated hsCRP. The significance of findings in this study and the relative importance of different pathophysiological processes in harmful and hazardous drinkers should be further investigated using heart imaging methods. Echocardiography and cardiac magnetic resonance imaging can give more information about significance of cardiomyopathy‐related indexes in excessive drinkers. Computerized tomography coronary angiogram or carotid ultrasound will allow direct measurement of the extent of atherosclerosis and will answer the question about significance of elevated hsCRP levels in harmful and hazardous drinkers.

The results of this study help to explain why heavy alcohol drinking has been related to excess mortality in Russia if considered in the context of previous studies exploring causes of high cardiovascular mortality in Russia.^31^ Public health researchers and practitioners need to take account of the cardiotoxic effects of heavy drinking in populations, even when the levels of diagnosed frank alcoholic cardiomyopathy may be relatively low.

## Sources of Funding

The Know Your Heart study is a component of the International Project on Cardiovascular Disease in Russia (IPCDR). IPCDR was funded by a Wellcome Trust Strategic Award (100217), supported by funds from the University in Tromsø The Arctic University of Norway; Norwegian Institute of Public Health; and the Norwegian Ministry of Health and Social Affairs. The funders had no role in study design, data collection and analysis, decision to publish, or preparation of the manuscript.

## Disclosures

None.

## Supporting information


**Table S1.** Association of High Values of hs‐cTrT (Above 9.34 ng/L) With Levels of Alcohol Use (Logistic Regression Analysis)
**Figure S1.** The directed acyclic graph (DAG) depicting the suggested causal relationship between heavy alcohol use and heart damage (hs‐cTrT serving as a biomarker).
**Figure S2.** The directed acyclic graph (DAG) depicting the suggested causal relationship between heavy alcohol use and cardiac wall stretch (NT‐proBNP serving as a biomarker).
**Figure S3.** The directed acyclic graph (DAG) depicting the suggested causal relationship between heavy alcohol use and systemic inflammation (hsCRP serving as a biomarker).
