# Supplementary material for: Evidence for a Direct Harmful Effect of Alcohol on Myocardial Health: A Large Cross‐Sectional Study of Consumption Patterns and Cardiovascular Disease Risk Biomarkers From Northwest Russia, 2015 to 2017
Source: J Am Heart Assoc. 2019 Dec 18;9(1):e014491. doi: 10.1161/JAHA.119.014491 (PMC6988140; doi:10.1161/JAHA.119.014491)

SUPPLEMENTAL MATERIAL

Table S1. Association of high values of hs-cTrT (above 9.34 ng/L) with levels of alcohol use (logistic regression analysis).

	Model 1 (adjusted for age and sex), OR (95% CI)	Model 2 (additionally adjusted for smoking and education), OR (95% CI)	Model 3 (additionally adjusted for other covariates), OR (95% CI)
**hsTrT**, N = 2595.			
**Alcohol use**			
Narcology clinic subsample	1.72 (1.22, 2.44)	2.01 (1.36, 2.97)	2.25 (1.5, 3.39)
General population sample, harmful drinkers	0.38 (0.17, 0.84)	0.46 (0.2, 1.03)	0.48 (0.21, 1.1)
General population sample, hazardous drinkers	0.91 (0.68, 1.23)	0.92 (0.68, 1.25)	0.92 (0.67, 1.26)
General population sample, non-problem drinking	1.0 [reference group]	1.0 [reference group]	1.0 [reference group]
General population sample, non-drinkers	1.03 (0.72, 1.47)	0.99 (0.69, 1.43)	0.89 (0.61, 1.3)
P-value for trend test (among drinkers), df=1	0.132	0.0734	0.032
P-value for heterogeneity test, df=4	< 0.001	< 0.001	< 0.001

Figure S1. The directed acyclic graph (DAG) depicting the suggested causal relationship between heavy alcohol use and heart damage (hs-cTrT serving as a biomarker).

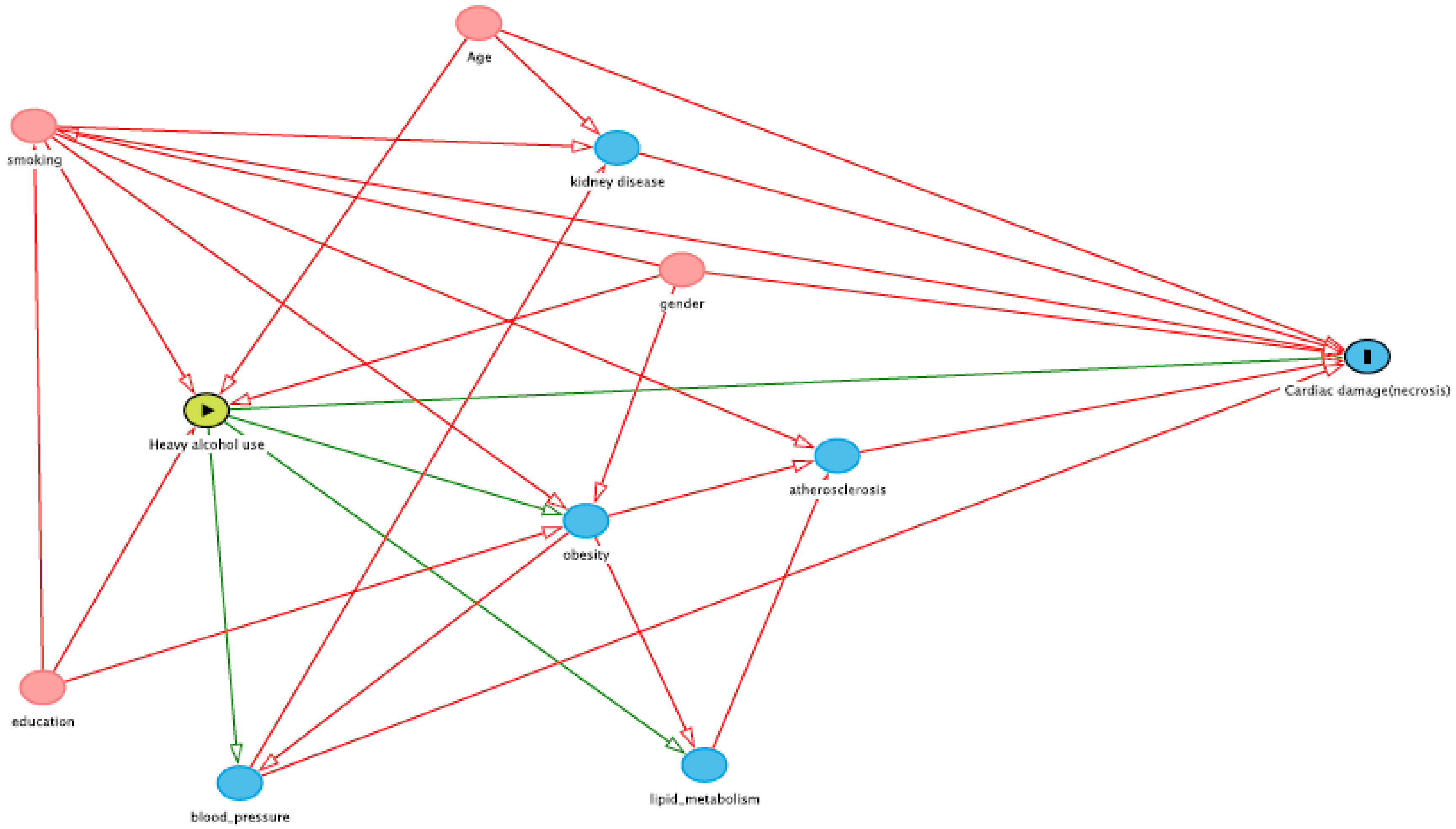

Figure S2. The directed acyclic graph (DAG) depicting the suggested causal relationship between heavy alcohol use and cardiac wall stretch (NT-proBNP serving as a biomarker).

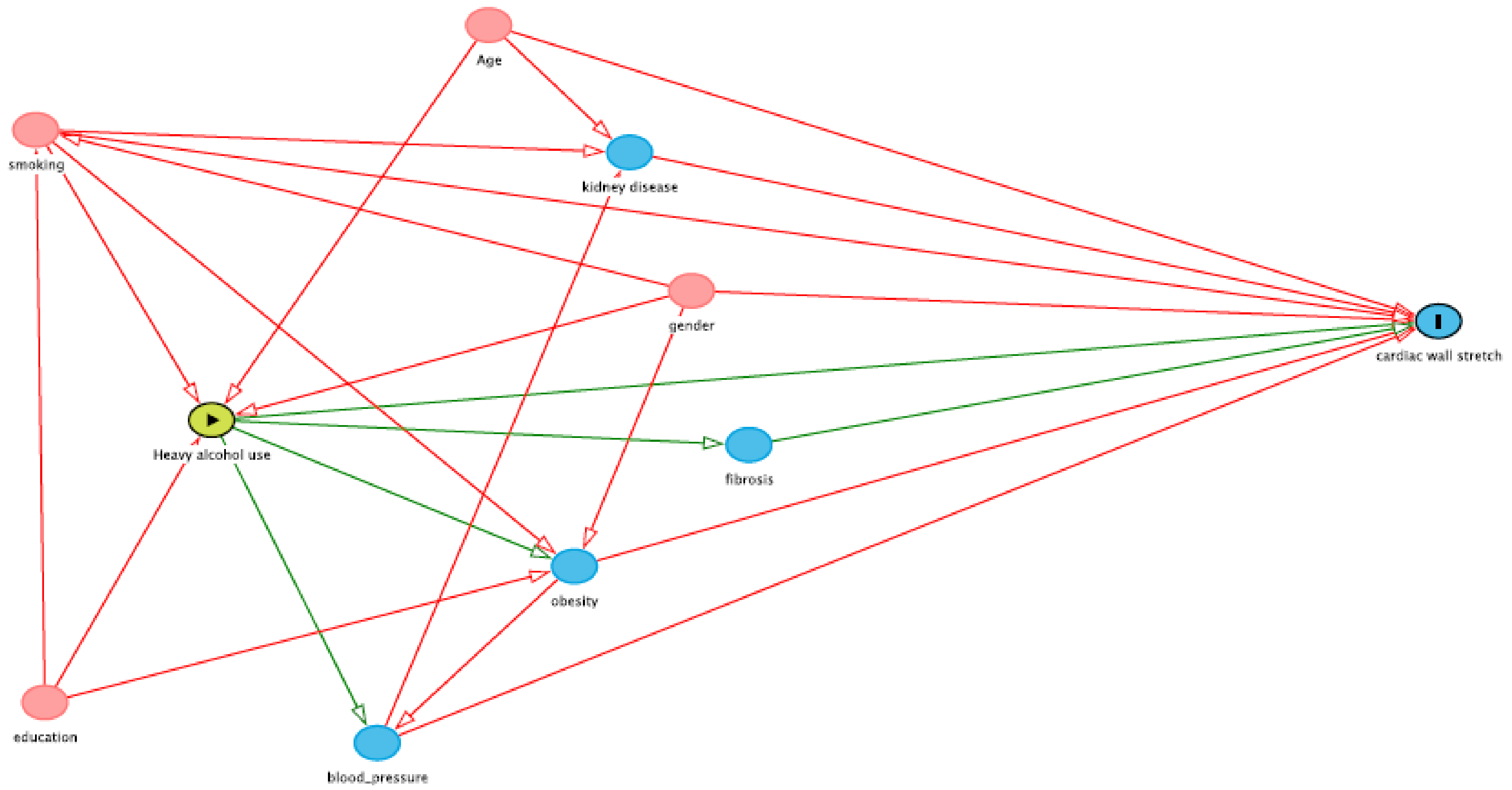

Figure S3. The directed acyclic graph (DAG) depicting the suggested causal relationship between heavy alcohol use and systemic inflammation (hsCRP serving as a biomarker).

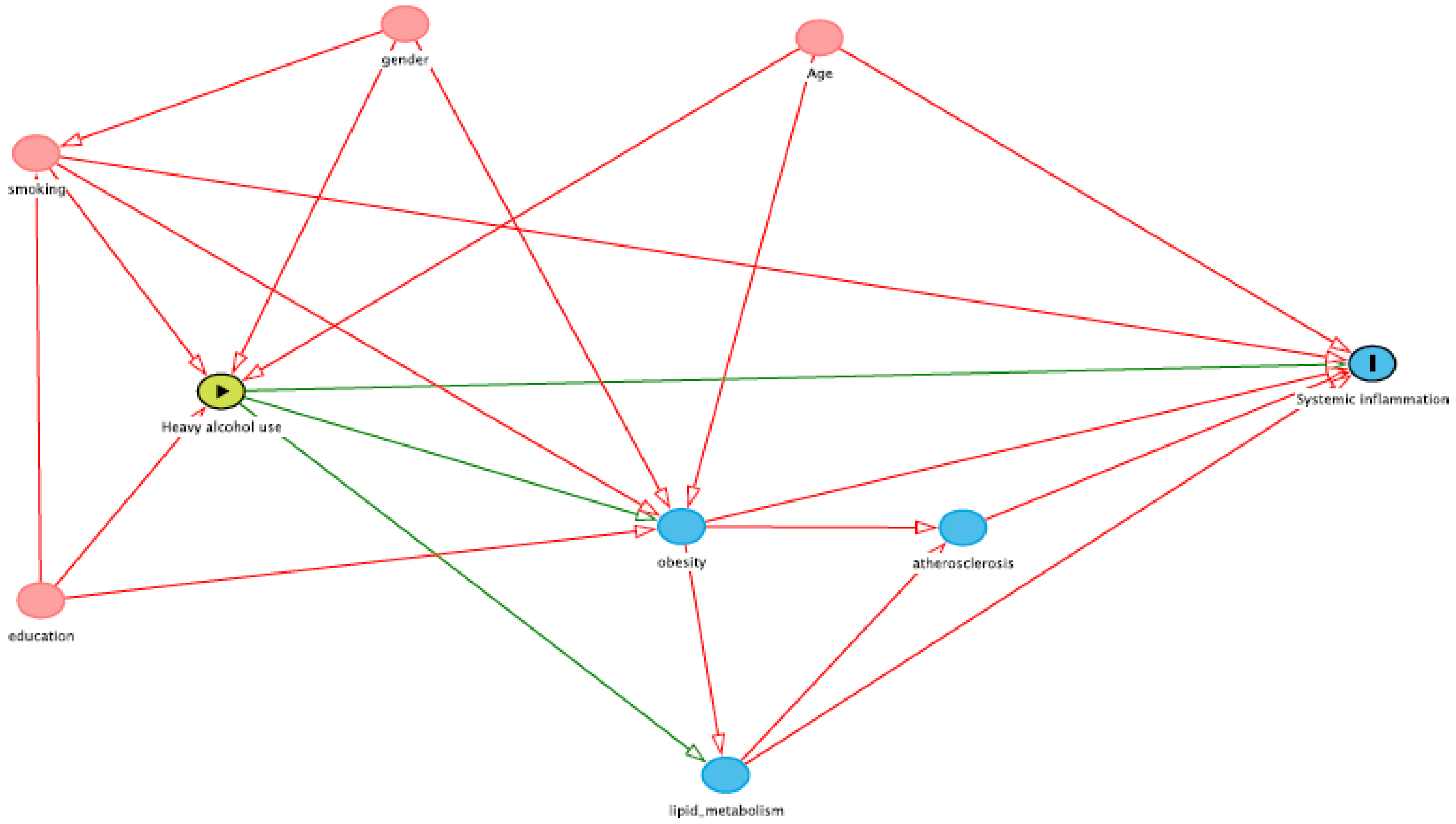

Supplement: Supplementary file 1 — Table S1. Association of High Values of hs‐cTrT (Above 9.34 ng/L) With Levels of Alcohol Use (Logistic Regression Analysis) Figure S1. The directed acyclic graph (DAG) depicting the suggested causal relationship between heavy alcohol use and heart damage (hs‐cTrT serving as a biomarker). Figure S2. The directed acyclic graph (DAG) depicting the suggested causal relationship between heavy alcohol use and cardiac wall stretch (NT‐proBNP serving as a biomarker). Figure S3. The directed acyclic graph (DAG) depicting the suggested causal relationship between heavy alcohol use and systemic inflammation (hsCRP serving as a biomarker). [file JAH3-9-e014491-s001.pdf]
